# Response to Ketamine Therapy in Anxious and Non-Anxious Major Depressive Disorder: A Meta-Analysis of Clinical Trials

**DOI:** 10.1192/j.eurpsy.2025.350

**Published:** 2025-08-26

**Authors:** I. Borja De Oliveira, A. L. Stephany, M. L. Geremias, D. Xavier, F. Wagner, A. L. Balduino de Souza, M. O. Pozzolo Pedro, D. Soler Lopes, M. C. Carbajal Tamez, J. Quevedo, M. Teranishi

**Affiliations:** 1 School of Medicine, University of São Paulo, São Paulo; 2 Integrated Medicine Service, Jacareí; 3 University of the Joinville Region, Joinville, Brazil; 4 ECPE - PPCR Program, Harvard T. H. Chan School of Public Health, Boston, United States; 5 School of Medicine, Pontifical Catholic University of Rio Grande do Sul, Porto Alegre; 6 Evangelical University of Goias, Anapolis; 7 Departament and Institute of Psychiatry, University of São Paulo, São Paulo, Brazil; 8 Lancaster University, Lancaster, United Kingdom; 9 Department of Psychiatry and Behavioral Sciences at McGovern Medical School; 10 Center for Interventional Psychiatry, Faillace Department of Psychiatry and Behavioral Sciences, McGovern Medical School, The University of Texas Health Science Center at Houston, Houston, United States; 11 Università degli Studi di Milano, Milan, Italy

## Abstract

**Introduction:**

Anxious depression (AxD) as an independent diagnostic has been controversial, with many suggesting it as a transient state and others highlighting evidence of a worse outcome, severity, and increased suicide risk. The International Classification of Diseases (ICD-11) lists a related concept under 6A73, Mixed depressive and anxiety disorder. Previous literature on ketamine’s efficacy has mainly focused on either anxiety or depression, with limited comparison of both groups. Given their high comorbidity and shared pathophysiology, we aimed to assess ketamine’s efficacy in these populations.

**Objectives:**

This meta-analysis aimed to consolidate evidence from clinical trials evaluating ketamine therapy in AxD and Non-Anxious Depression (NAxD).

**Methods:**

A search for published clinical trials in indexed journals and databases was conducted on August 11, 2024. Keywords included ketamine, anxiety, comorbidity, and depression, with no restrictions on language or publication date. Studies on bipolar or psychotic depression were excluded. A random-effects model accounted for variability, and subgroup analyses were performed.

**Results:**

Eight studies involving 536 participants (mean age = 39.0 years) were preselected. Seven studies defined “anxious depression” as a score of 7 or higher on the HAMD-AS, with AxD mean of 8.74 (±0.56) and NAxD mean of 5.83 (±1.9). MADRS scores were 35.18 (±2.22) for AxD and 31.97 (±2.29) for NAxD. The effect size of improvement in depressive symptom severity (as assessed by the MADRS) was not significantly different between the groups either 13 days after treatment (SMD = -0.07[-0.69, 0.55], p = 0.82, I2 = 73%) or 26-28 days after treatment (SMD = -0.30[-0.64, 0.04], p = 0.09, I2 = 21%). The overall depression response also did not significantly differ between the groups (odds ratio = 0.84 [0.50, 1.41], p = 0.52, I² = 13%). Insufficient data were available for remission rates.

**Conclusions:**

Ketamine shows comparable efficacy in reducing depressive symptoms and achieving response in both groups. The group classified as AxD parallels previous reports of increased severity when reviewing baseline scores MADRS and other available scores. Thus, ketamine should be considered a viable treatment for patients with AxD, as they may have lower response rates to traditional antidepressants. This analysis was limited by the small number of studies, small sample sizes, and moderate heterogeneity. Differences in baseline depressive symptom severity and varying definitions of MDD with anxiety also constrained our analysis. Given the severity of symptoms in this population, we recommend developing better classification instruments for AxD. Further research is needed to explore remission differences in AxD and refine treatment strategies.

**Disclosure of Interest:**

I. Borja De Oliveira: None Declared, A. Stephany: None Declared, M. Geremias: None Declared, D. Xavier: None Declared, F. Wagner: None Declared, A. Balduino de Souza: None Declared, M. O. Pozzolo Pedro: None Declared, D. Soler Lopes: None Declared, M. Carbajal Tamez: None Declared, J. Quevedo Shareolder of: Instituto de Neurociencias Dr. Joao Quevedo, Grant / Research support from: LivaNova; and receives copyrights from Artmed Editora, Artmed Panamericana, and Elsevier/Academic Press, Consultant of: EMS, Libbs, and Eurofarma, Speakers bureau of: Myriad Neuroscience and AbbVie., M. Teranishi: None Declared

